# Electroosmotic flow driven microfluidic device for bacteria isolation using magnetic microbeads

**DOI:** 10.1038/s41598-019-50713-z

**Published:** 2019-10-02

**Authors:** Samuel Miller, Alison A. Weiss, William R. Heineman, Rupak K. Banerjee

**Affiliations:** 1 Department of Mechanical and Materials Engineering, University of Cincinnati, 598 Rhodes Hall, University of Cincinnati, Cincinnati, OH 45221 USA; 2 Department of Molecular Genetics, Biochemistry & Microbiology, University of Cincinnati, 2254 Medical Sciences Building, 231 Albert Sabin Way, Cincinnati, OH 45267 USA; 3 Department of Chemistry, University of Cincinnati, 120 Crosley Tower, PO Box 210172, Cincinnati, OH 45221 USA; 4 Department of Mechanical and Materials Engineering, University of Cincinnati, 593 Rhodes Hall, ML 0072, University of Cincinnati, Cincinnati, OH 45221 USA

**Keywords:** Mechanical engineering, Sensors and biosensors

## Abstract

The presence of bacterial pathogens in water can lead to severe complications such as infection and food poisoning. This research proposes a point-of-care electroosmotic flow driven microfluidic device for rapid isolation and detection of *E. coli* in buffered solution (phosphate buffered saline solution). Fluorescent *E. coli* bound to magnetic microbeads were driven through the microfluidic device using both constant forward flow and periodic flow switching at concentrations ranging from 2 × 10^5^ to 4 × 10^7^ bacteria/mL. A calibration curve of fluorescent intensity as a function of bacteria concentration was created using both constant and switching flow, showing an increase in captured fluorescent pixel count as concentration increases. In addition, the use of the flow switching resulted in a significant increase in the capture efficiency of *E. coli*, with capture efficiencies up to 83% ± 8% as compared to the constant flow capture efficiencies (up to 39% ± 11%), with a sample size of 3 µL. These results demonstrate the improved performance associated with the use of the electroosmotic flow switching system in a point-of-care bacterial detection assay.

## Introduction

The development of micro total analysis systems (µTAS), often referred to as lab-on-chip devices, have ever evolving technological requirements to overcome the difficulties inherent in performing rapid biological assays in a small portable device^1^. The µTAS devices are designed to utilize micro- to nano-liter samples while still providing reliable and swift analysis for point-of-care diagnostics. The difficulty of using these devices in a biological assay stems from the need to downscale laboratory process, such as biomolecular targeting, spectroscopy, and image analysis, that requires large equipment and long wait times, into a portable and quick-response system^2^. Typical bacterial toxicity levels range from 10^6^ to 10^8^ bacteria/mL, with some particularly infectious strains reporting infectious doses of less than 1000 bacteria/mL^3^.

Magnetophoretic based immunoassay is a popular separation technique used in µTAS devices. This technique utilizes magnetic microbeads (mMBs) coated with binding ligands that will bind to specific biomolecules so that the mMB-complex can be isolated in the system^4–7^. This separation is accomplished by applying a magnetic field to the system to isolate, or capture, the mMBs and, therefore, the biomolecules that are bound to them. A high capture efficiency, or the ratio between captured beads and the total beads in the system, is essential to the minimization of error in a microfluidic device. The use of mMBs is ideal for this application due to the ease at which they are isolated in the system using the external magnetic field, their high surface area-to-volume ratio that allows for availability of ample binding sits, and their ability to be adapted to target a multitude of different biomolecules^8–10^. These factors make magnetophoretic separation using mMBs a promising choice for use in a µTAS device that is both reliable and versatile in its application.

### Literature review and background

A limited number of studies have been done examining the use of magnetophoretic separation of mMBs. Thompson *et al*. (2010) studied how the incorporation of microwells affect capture of mMBs in a microfluidic channel, reporting a low capture efficiency of around 13%^11^. A study by Di Carlo *et al*. (2007) aims to use inertial focusing in the microchannel in an attempt to improve capture efficiency^12^. This process uses a series of s-shaped bends in a microchannel that organize the mMBs into a single flow line to make them easier to isolate at the outlet. Li *et al*. (2013) further evaluated how adjusting streamlines in a channel by adding obstacles in the flow path affect capture of particles^13^. Additional changes in channel geometry were part of a numerical study that aimed at filtering based on particle size by Zhang *et al*. (2014)^14^. A numerical study by Wu *et al*. (2011) attempted to predict how capture efficiency changes between a straight channel, L-shaped channel, and T-shaped channel^15^. A study by Beyor *et al*. (2008) used special pumps to create a pulsatile flow in the channel^6^. Using these pumps, the fluid was driven back and forth through the channel, showing improved capture efficiency up to 70% due to the multiple passes.

In addition to analysis of channel geometry and flow, how changes in magnetic field in the channel affects mMB capture has also been studied. Munir *et al*. (2009) performed a numerical study analyzing how the variation of magnet position and number of magnets around a circular chamber in a microchannel affect the capture efficiency of mMB^16^. A study by Hoshino *et al*. (2011) explored alternating magnetic polarities along a channel in an attempt to separate mMBs and cancer cells in blood samples^17^. Ramadan *et al*. (2009) examined whether the use of electromagnets allows for better control and isolation of mMBs^18^. However, this study created the microchannel using a silicon wafer instead of transparent glass slide while using a high-pressure syringe pump to drive the flow.

The previous work on mMB capture in microfluidic channels has been performed on a few different channel and magnet configurations. However, most of the studies were numerical in nature while the majority of the experimental studies utilize pressure driven flow instead of electroosmotic flow (EOF) to drive the fluid through the channel. The use of pumps on such smaller scales is expensive and complicated. In addition, the extremely high pressure necessary to pump the fluid through the small microchannels often causes leaking at the junction between the channel and the pump as well as seepage at the bonds between the polymer and glass slide.

The isolation of pathogens in samples using various methods has also been analyzed. Kwon *et al*. (2008) used streptavidin coated mMBs tagged to fluorescent antibodies to detect toxins in a system^7^. Faridi *et al*. (2017) analyzed how inertial effects based on particle size affect the separation of bacteria from blood samples in a microchannel, reporting separation efficiencies up to 76% and a processing rate of 0.5 µL/min^19^. A study by Li *et al*. (2017) analyzed how external acoustic effects applied to a microchannel contribute to bacterial separation from blood in the channel^20^. They used digital transducers to apply acoustic waves to the system and form pressure nodes in the channel that separate *E. coli* from the red blood cells due to the cells’ physical properties. They reported a resulting blood purity of 96%^20^. A study by Wang *et al*. (2012) used antibody coated channels and an incubation time of 30 minutes to identify fluorescent *E. coli*, with capture efficiencies of 71.8%^21^. The majority of the bacterial separation work using microchannels does not use any magnetic separation techniques. Instead, these studies opt for separation techniques that do not require the target entity to be bound to mMBs. While this strategy removes a step in the sample preparation process, it limits the specificity and adaptability of the separation process. The use of mMBs allows for the targeted capture of a specific bacterial species and differentiation between multiple types of bacteria in a sample.

### Proposed design

Efficient capture of mMBs and ability to be selective in bacteria targeting are important for the sensitivity and effectiveness of any proposed lab-on-chip device. Our group has developed a µTAS device that uses electroosmotic flow (EOF) and fluorescent microscopy to capture mMBs and quantify their concentration in a microfluidic system. The use of EOF is preferred to pressure driven flow due to it being inexpensive and efficient when operating at small volumes while providing improved control over flow rate and direction^22,23^. The developed µTAS device takes advantage of EOF by using a flow switching system to increase capture efficiency (η_c_) by returning uncaptured beads to the area of the channel with higher magnetic field strength. This device has shown capture efficiencies of fluorescent mMBs (mMB* complexes) under switching flow of up to 85% while analyzing concentrations as low as 10^6^ beads/mL^24^.

The previous study only analyzed mMB*s in the system^24^, while this study takes the next step in developing the system for bacterial analysis under realistic scenarios. This work is a *novel* extension on the previous study due to the introduction of the bacterial pathogen as the fluorescent entity of interest. The previously built device is used to capture and identify magnetic microbead-fluorescent bacteria (mMB-E. coli*) complexes under realistic test scenarios using very small sample volumes. This research is *significant* because it will allow the assessment of capture efficiency for screening different types of water-borne pathogens while requiring a much smaller sample volume (around 30% volume) than other devices reported in literature. It was *hypothesized* that the devices would be able to effectively capture mMB-E. coli* complexes, while allowing for: (1) the creation of a calibration curve of fluorescent intensity as a function of a wide range of mMB-E. coli* concentration and (2) comparison of capture efficiency for constant and switching flows. This would prove the usefulness of the system as a point-of-care µTAS device for high-throughput screening of pathogens.

## Methods

This section describes the methods used to create the device, the materials used, the design of the experiment, and the analysis performed. In the experiments, mMB-secondary antibody-fluorescent bacteria complexes (mMB-E. coli*) were injected into a PDMS microchannel and driven through the channel using EOF. The mMB-E. coli* complexes were immobilized in the channel using an external magnet and the fluorescence was characterized using inverted fluorescent microscopy. The captured images were analyzed using MATLAB to determine the captured and uncaptured fluorescent intensities. The capture efficiency was calculated for both switching and constant flow protocols. These protocols were compared based on relative percentage difference, and a calibration curve was created based on total fluorescent intensity.

### Material properties

**Figure 1:**
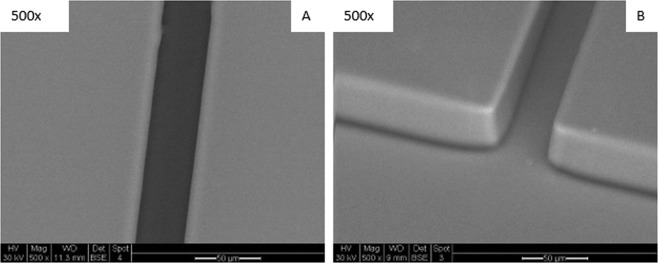
SEM images of (**A**) a top down view of the microchannel and (**B**) a tilted view of the entrance to the microchannel from a well.

**Figure 2:**
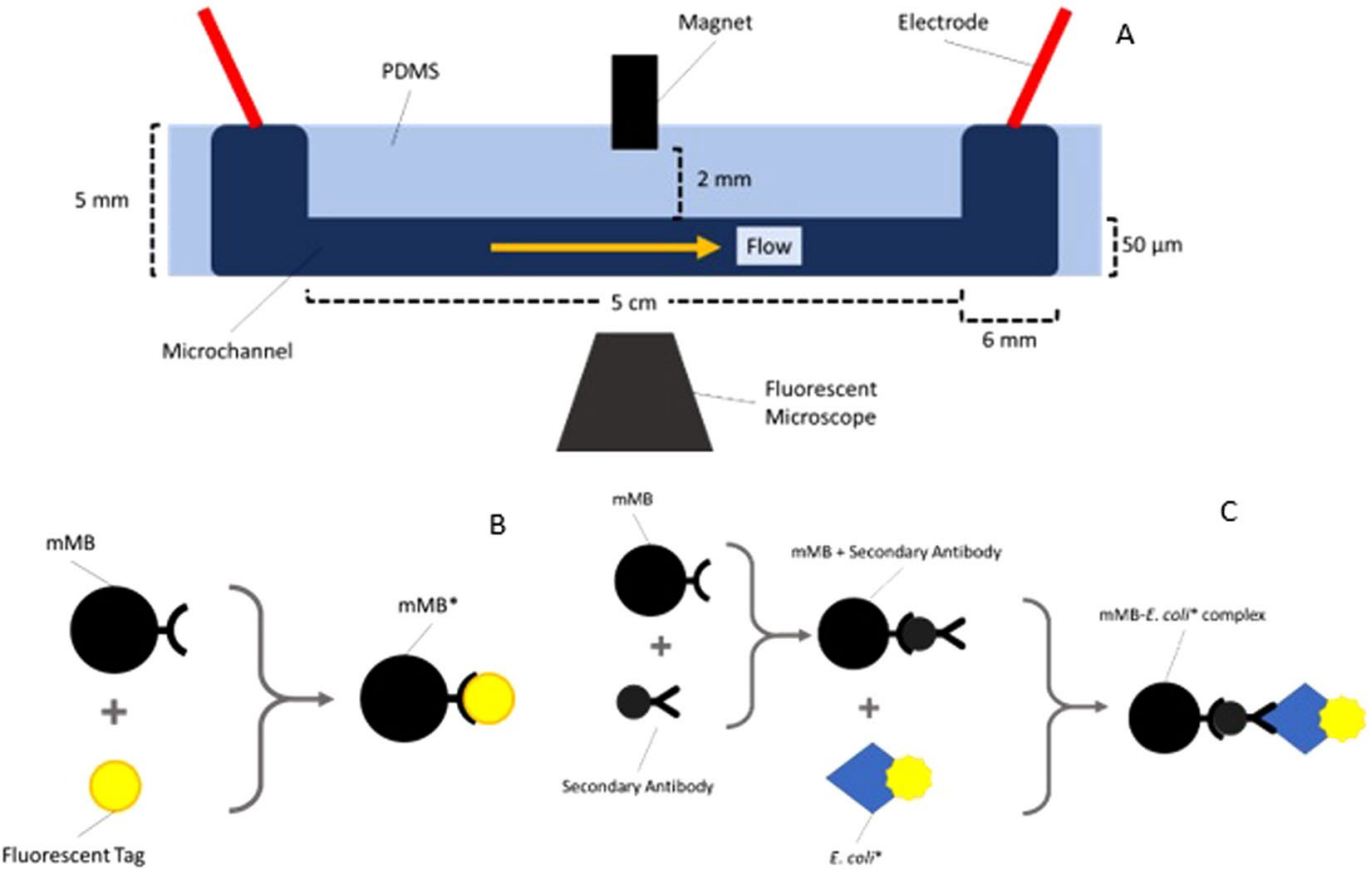
(**A**) Schematic of the microchannel testing system, (**B**) schematic of the mMB* system^24^, and (**C**) schematic of the mMB-E. coli* system used for *E. coli* capture.

The microfluidic device was created following the protocol outlined in the previous publication^24^. In brief, the device was made of PDMS bound to a glass slide to create a 50 mm long microchannel with a 50 µm × 50 µm cross section with wells at each end of the channel. SEM images of the PDMS channel and one of the wells are shown in Fig. 1A,B. A 1/8″ × 1/8″ × 3/8″ volume neodymium (NdFeB) magnet was placed above the microchannel halfway between the two wells. A diagram of the experimental setup is shown in Fig. 2A.

The mMBs used in the experiments were 2.8 µm diameter Dynabead M280 Sheep anti-Rabbit IgG mMBs (Life Technologies, NY). These mMBs are bound to inherently fluorescent *E. coli* (E. coli*) by a secondary Virustat anti-*E. coli* rabbit polyclonal antibody. A schematic of the fluorescent mMB complex (mMB*) without *E. coli* is shown in Fig. 2B whereas a schematic of the new mMB-E. coli* complex is shown in Fig. 2C. The mMB* complexes were created by binding M280 sheep anti-rabbit Dynabeads with Alexa Fluor 488 rabbit anti-mouse fluorescent tag following the procedure described in the previous publication^24^. The mMB-E. coli* complexes were created by incubating 0.4 mL of E. coli*, with a concentration of about 6 × 10^8^ bacteria/mL, with 0.6 mL of Dynabeads, with a concentration of 7 × 10^8^ beads/mL, and excess secondary antibody by slow tilt mixing for 24 hours at 8 °C. The mMB-E. coli* solution was then diluted with phosphate buffer (PBS, pH 7.5) to achieve the desired concentration for each experiment.

### Experimental method

To ensure adequate flow when the external voltage was applied, the channels were treated with 1 M NaOH solution for 10 minutes. The channel was then washed with PBS buffer and, prior to each experiment, was primed with a 1% Tween 20 in PBS buffer solution for 5 minutes to decrease the surface tension and prevent the mMBs from sticking to the channel walls while not captured.

After channel preparation, the mMB-E. coli* solution was injected into the inlet well and the chip was placed on the inverted fluorescent microscope. The magnet was put in place and two platinum electrodes were connected to a high voltage sequencer (LabSmith, CA) and placed into the inlet and outlet wells. The experiments were performed under two previously published flow conditions, a switching flow protocol and a constant flow protocol^24^, which are described in further detail below. Under both protocols, the flow was driven at 650 V EOF voltage. The microscope captured images using MetaMorph Software (Molecular Devices, PA).

The 650 V flow rate is used to determine the maximum distance before and after the magnet where mMBs are captured. To determine the capture zone, samples were driven through the channel at the lower limit of the voltage. Immediately following the experimental time, the channel was washed with pure PBS buffer with the magnet still in place. This removed any uncaptured mMBs from the channel so that the maximum distance before and after the magnet that contained mMBs was determined to be the capture zone. Thus, when performing experiments with mMB-E. coli* complexes, any mMB-E. coli* found in the capture zone are considered captured while those mMB-E. coli* complexes that are found after the capture zone or in the outlet well are considered uncaptured. The mMB-E. coli* complexes that reside in the inlet well or in the channel before the capture zone are not considered as they never entered the zone of magnetic field generated by the stationary magnet and, thus, never entered the experiment.

### Characterization

The purpose of this device is to identify mMB-E. coli* concentration in an unknown sample, thus the development of a calibration curve for the device is required. The calibration curve plots total fluorescent intensity as a function of concentration. The total fluorescent intensity of a sample with unknown mMB-E. coli* complex concentration can be measured and compared to the curve to determine the mMB-E. coli* concentration. The total fluorescent intensity is determined by the sum of the fluorescent intensities of all captured mMB-E. coli* complexes in a single experiment. In addition, the *capture efficiency* (η_c_) and *relative percentage difference* between the constant and switching capture efficiencies were calculated. The capture efficiency (η_c_) was calculated according to Eq. (1) and is based on the ratio of the captured mMB-E. coli* complexes to the total number of mMB-E. coli* complexes that entered the channel based on fluorescent pixel count.

1 $${{\rm{\eta }}}_{{\rm{c}}}=\frac{{\rm{pixel}}\,{\rm{count}}\,{{\rm{captured}}}_{{\rm{mMB}}-E.coli\ast }}{{\rm{pixel}}\,{\rm{count}}\,{{\rm{captured}}}_{{\rm{mMB}}-E.coli\ast }+{\rm{pixel}}\,{\rm{count}}\,{{\rm{uncaptured}}}_{{\rm{mMB}}-E.coli\ast }}$$

The relative percentage difference between the constant and switching capture efficiency at each concentration was calculated according to Eq. (2),

2 $$Relative\, \% \,Difference=\frac{{\eta }_{c\_switching}-{\eta }_{c\_constant}}{{\eta }_{c\_constant}}\times 100 \% $$

Statistical analysis was then performed using a t-test, where p < 0.05 is considered statistically significant.

### Constant flow and switching flow protocols

**Figure 3:**
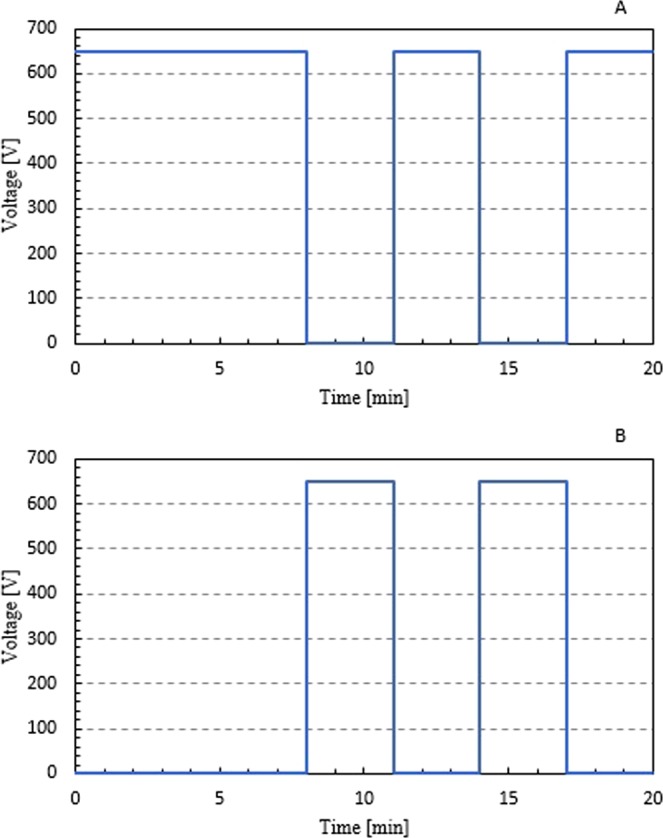
(**A**) Inlet voltage as a function of time and (**B**) outlet voltage as a function of time for the switching flow protocol.

The switching flow protocol experiments are performed under 650 V EOF flow for 8 minutes forward followed by two periods of alternating 3 minutes backward and then 3 minutes forward for a 20 minute total testing time. The voltages as a function of time at the inlet and outlet of the channel for the switching flow profile are shown in Fig. 3A,B, respectively. The flow rate during one of these periods can be calculated according to the Helmholtz-Smoluchowski equation, as shown in Eq. (3),

3 $${{\rm{U}}}_{{\rm{ep}}}=-\frac{{{\rm{E}}}_{{\rm{z}}}{{\rm{\varepsilon }}}_{{\rm{r}}}{{\rm{\varepsilon }}}_{{\rm{o}}}{{\rm{\zeta }}}_{{\rm{p}}}}{{\rm{\mu }}}$$

where U_ep_ is the velocity (cm/s), E_z_ is the applied electric field (V/cm), ε_r_ is the dielectric constant of the medium, ε_o_ is the vacuum permittivity (F/m), ${{\rm{\zeta }}}_{{\rm{p}}}$ is the zeta potential (V), and μ is the dynamic viscosity (Pa·s). For the 650 V case, E_z_ is 130 V/cm, ε_r_ is 80.4, ε_o_ is 8.55 × 10^−12^ F/m, ${{\rm{\zeta }}}_{{\rm{p}}}$ is −95.6 mV, and μ is 8.6 × 10^−4^ Pa·s [Das *et al*. 2016]. The fluid velocity at this voltage was determined to be 0.099 cm/s, which equates to a volumetric flow rate of 0.15 µL/min. For a 20 minute testing period, this system will process a 3 µL sample. With a 5 cm long channel, a 3 minute period would allow for about 3.5 full channel length (5 cm) clearances. Thus, this EOF voltage provides ample time for multiple passes of mMB-E. coli* complexes through the channel during each testing period.

During the 20 minute switching flow test, the first 8 minutes of forward flow will introduce the vast majority of mMB-E. coli* complexes to the system, since the subsequent reversals of flow will alternate these mMB-E. coli* complexes flowing backward and forward. Therefore, in order to better compare the total fluorescent intensities between the constant and switching flow protocols for the calibration curve, an 8 minute testing time was used for the constant flow protocol. The 8 minute constant flow time corresponds to the 8 minutes of forward flow for the switching protocol since the oscillating portion of the switching flow protocol is primarily used to increase the residence time of the mMB-E. coli* complexes in the area of higher magnetic field strength within the channel. This switching allows complexes that may have escaped capture on the first pass enough time to travel the transverse distance along the cross section of the channel and be captured.

## Results

The microfluidic device was tested using mMB-E. coli* complexes concentrations of 2 × 10^5^, 2 × 10^6^, 4 × 10^6^, 2 × 10^7^, and 4 × 10^7^ bacteria/mL under switching and constant flow protocols at an EOF voltage of 650 V. Fluorescent images were taken to determine a calibration curve and the capture efficiency based on fluorescent intensity of mMB-E. coli* complexes.

### The mMB-E. coli* tagging

**Figure 4:**
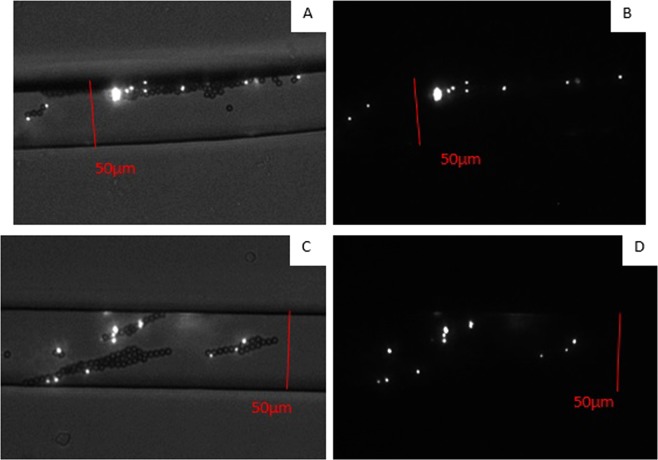
Fluorescent images that captured mMB-E. coli* complexes with (**A**) and (**B**) showing a different area and (**C**) and (**D**) showing the same area. (**A**) and (**C**) are taken using the external light source to show both the E. coli* and the attached mMBs, while (**B**) and (**D**) are taken under the dark condition used during experiments showing only the E. coli* fluorescence. The width of the channel is ~50 µm.

Solutions with mMB-E. coli* complexes concentrations of 2 × 10^5^, 2 × 10^6^, 4 × 10^6^, 2 × 10^7^, and 4 × 10^7^ bacteria/mL were tested in a microfluidic device using both the constant and switching flow protocols at an EOF voltage of 650 V. Fluorescent images were taken along the length of the channel for each concentration, with images in the capture zone determined to be captured and images after the capture zone counted as uncaptured. In order to ensure that mMB-E. coli* complexes were forming and that the E. coli* were the only fluorescent entity in the system, images were taken of the same area with and without an additional light source. This light source shines white light on the channel along with the exciting laser required for fluorescence. This allows viewing of the fluorescent entity while also illuminating the mMBs in the system via the white (secondary) light. Figure 4 shows sample taken in the channel with and without this light source. Figure 4A,B show the same area of the channel with and without the white light source whereas Fig. 4C,D show a different area with and without the white light source. These comparative images demonstrate that any unbonded mMBs will not affect the laser (primary light) induced fluorescent images in the condition without secondary white light used in the experiments. This is because the E. coli* portion of the mMB- E. coli* complexes are the only (primary) fluorescent entities.

### Calibration curve

**Figure 5:**
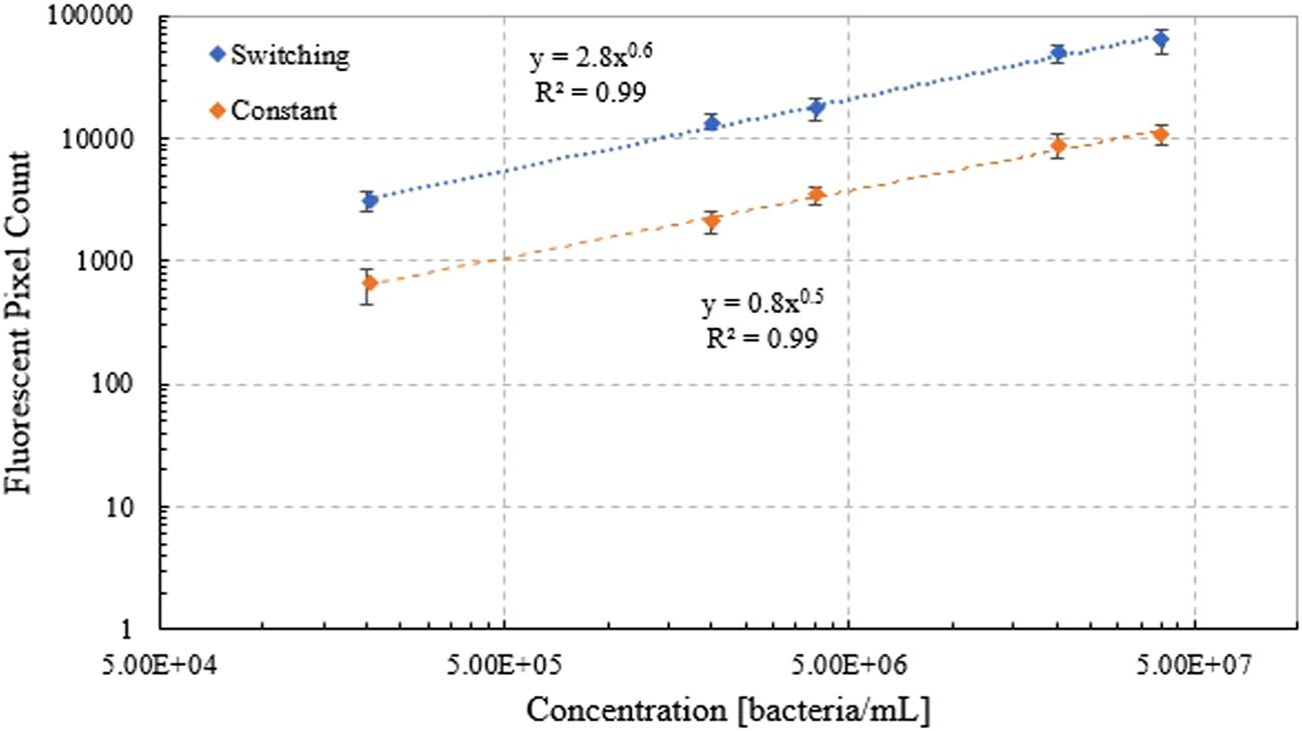
Calibration curve of fluorescent intensity as a function of concentration for capture of mMB-E. coli* complexes in a microchannel under the switching flow protocol at 650 V on log-log scale plot.

**Figure 6:**
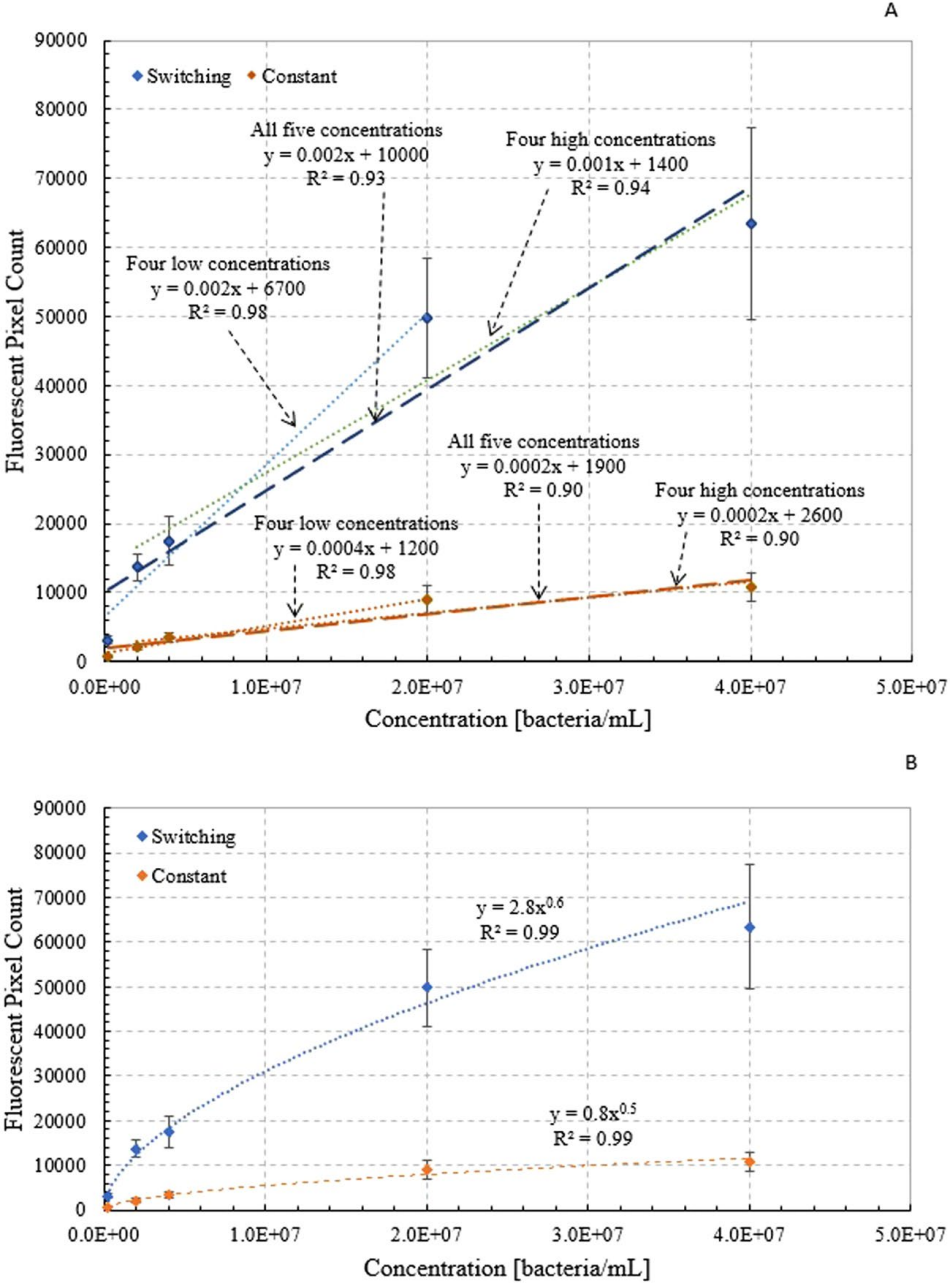
Calibration curve of fluorescent intensity of captured mMB-E. coli* complexes as a function of concentration in the microfluidic device under the constant flow protocol and EOF voltage of 650 V with standard scale axes using a (**A**) series of linear trendlines and (**B**) power law fit.

The mMB-E. coli* complexes were analyzed in the device to create a calibration curve of fluorescent intensity as a function of mMB-E. coli* concentration. The mMB-E. coli* complexes were delivered through the system at concentrations of 2 × 10^5^, 2 × 10^6^, 4 × 10^6^, 2 × 10^7^, and 4 × 10^7^ bacteria/mL and an EOF voltage of 650 V. For this analysis, only the images of captured mMB-E. coli* complexes were used to determine the fluorescent intensity. The calibration curve, shown in Fig. 5, shows fluorescent pixel count as a function of concentration on a log-log plot. Figure 6A,B show the calibration curve on a standard axes plot, with a series of linear fits shown in Fig. 6A and a power law fit shown in Fig. 6B. Both the switching and constant flow protocols result in a linearly increasing trendline on the log-log plot, with R^2^ values of 0.99 for both the switching and constant cases. There is a significant difference between the fluorescent intensities at each concentration for both the switching and constant flow protocols with relative differences ranging between 3.7 times $$(e.g.,\frac{3070-660}{660}\times 100 \% $$ $$for\,concentration\,of\,2\times {10}^{5}\,bacteria/mL)$$ to 5.6 times $$(e.g.,\frac{13560-2070}{2070}\times 100 \% \,for\,concentration\,of\,2\times {10}^{6}$$ $$bacteria/mL)$$ the constant flow values, and p < 0.05 for each case. For the constant flow protocol curve, p < 0.05 when comparing fluorescent intensity values for neighboring concentrations, except when comparing the fluorescent pixel count between 2 × 10^7^ and 4 × 10^7^ bacteria/mL (p ≈ 0.35). This is also the case when comparing neighboring concentrations on the switching flow curve except when comparing the fluorescent pixel count between 2 × 10^6^ and 4 × 10^6^ bacteria/mL (p ≈ 0.15), and then also between 2 × 10^7^ and 4 × 10^7^ bacteria/mL (p ≈ 0.22).

### Comparison between constant and switching flow of mMB-E. coli*

**Figure 7:**
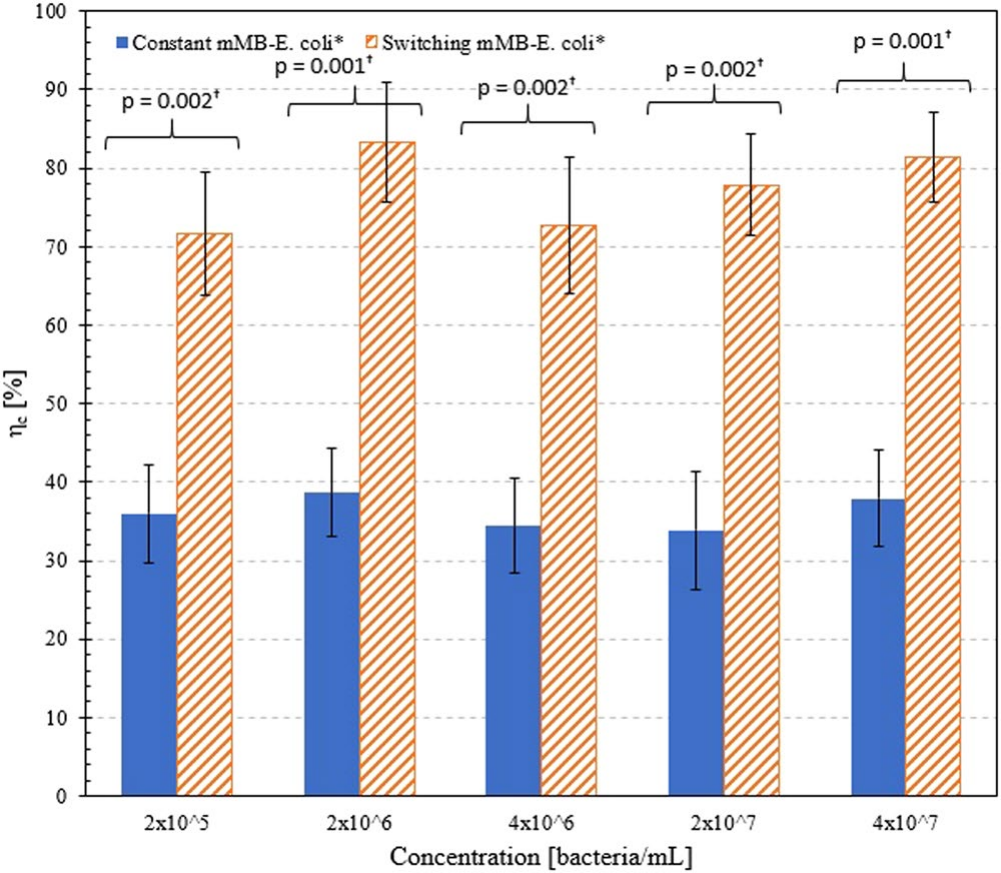
Capture efficiency of mMB-E. coli* complexes under switching flow based on fluorescent pixel counts at 650 V. (^**†**^ indicates statistical significance, p < 0.05).

The capture efficiency (η_c_) and relative percentage difference for mMB-E. coli* between the constant and switching cases at each concentration were also determined. The η_c_ were compared for mMB-E. coli* complexes at concentrations of 2 × 10^5^, 2 × 10^6^, 4 × 10^6^, 2 × 10^7^, and 4 × 10^7^ bacteria/mL under both constant and switching flow protocols, as shown in Fig. 7, with p < 0.05 at all concentrations. The values for captured, uncaptured, and total pixel count as well as the capture efficiency for the constant and switching cases are reported in Tables 1 and 2, respectively. The η_c_ for mMB-E. coli* complexes that are reported are calculated by averaging the capture efficiency values under the individual flow conditions. The η_c_ under the constant flow protocol were overall lower (compared to switching flow case; see below) at 36%, 39%, 34%, 34%, and 38% for concentrations of 2 × 10^5^, 2 × 10^6^, 4 × 10^6^, 2 × 10^7^, and 4 × 10^7^ bacteria/mL. In contrast, the switching flow protocol η_c_ were overall significantly higher (compared to constant flow case; see above) at 72%, 83%, 73%, 78%, and 81%for the same concentrations. The relative difference in η_c_ between switching and constant flows were about 2 times $(e.\,g.\,,\frac{72-36}{36}\times 100 \% \,at\,concentrations\,of\,2\times {10}^{5}\,bacteria/mL)$ for all mMB-*E*. coli* concentrations.

**Table 1:** Average capture efficiencies and pixel counts for the *constant flow* cases. The reported capture efficiency is calculated from the average of the individual capture efficiencies.

Conditions	Captured Pixel Count ± SD	Uncaptured Pixel Count ± SD	Total Pixel Count ± SD	Capture Efficiency (η_c_) ± SD
2 × 10^5^	660 ± 210	1150 ± 150	1810 ± 180	36 ± 9%
2 × 10^6^	2070 ± 430	3500 ± 1400	5570 ± 1570	39 ± 11%
4 × 10^6^	3440 ± 620	6810 ± 2050	10250 ± 1680	34 ± 10%
2 × 10^7^	8920 ± 2060	18010 ± 6050	26940 ± 6050	34 ± 9%
4 × 10^7^	10730 ± 2020	18320 ± 5810	29050 ± 4980	38 ± 11%

**Table 2:** Average capture efficiencies and pixel counts for the *switching flow* cases. The reported capture efficiency is calculated from the average of the individual capture efficiencies.

Conditions	Captured Pixel Count ± SD	Uncaptured Pixel Count ± SD	Total Pixel Count ± SD	Capture Efficiency (ηc) ± SD
2 × 10^5^	3070 ± 580	1290 ± 700	4360 ± 1270	72 ± 8%
2 × 10^6^	13560 ± 1890	2860 ± 1760	16510 ± 3030	83 ± 8%
4 × 10^6^	17500 ± 3610	6440 ± 1740	23940 ± 2350	73 ± 9%
2 × 10^7^	49850 ± 8670	13860 ± 3290	63710 ± 5960	78 ± 6%
4 × 10^7^	63430 ± 13980	13970 ± 2650	77410 ± 11950	81 ± 6%

### Comparison between mMB* and mMB-E. coli for all flow protocols

**Figure 8:**
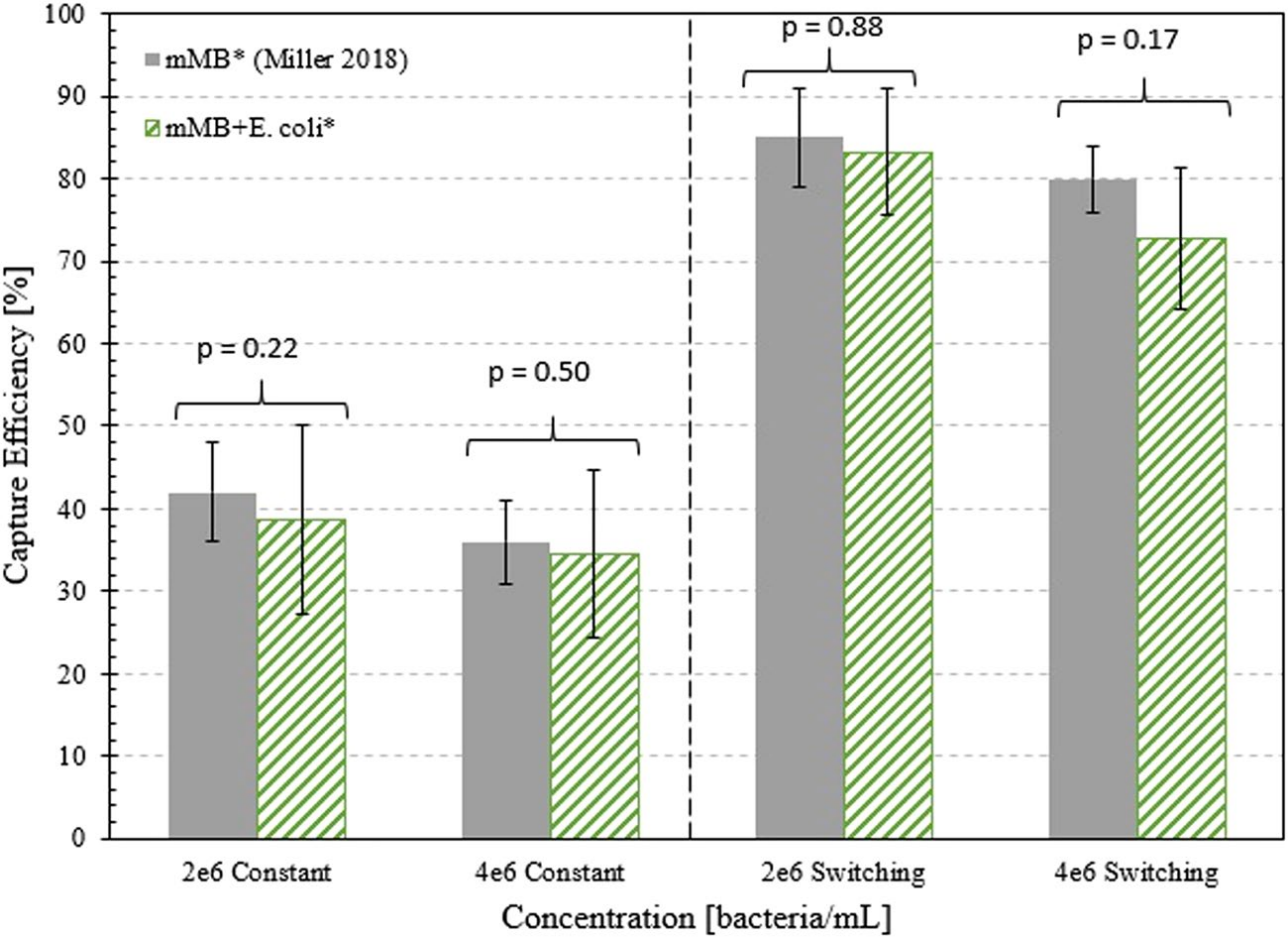
Comparison of capture efficiency of mMB* complexes^24^ and mMB-E. coli* complexes under constant and switching flow 2 × 10^6^.

The η_c_ of mMB-E. coli* complexes for constant and switching flow protocols at 2 × 10^6^ and 4 × 10^6^ bacteria/mL were compared to the η_c_ of only mMB* complexes (without *E. coli*)^24^, as shown in Fig. 8. For the comparison between η_c_ of mMB* and mMB-E. Coli* complexes at concentrations of 2 × 10^6^ bacteria (or bead for mMB* complex η_c_) /mL under constant flow, a η_c_ of 39% was reported for the mMB-E. coli* complexes while a η_c_ of 42% was reported for the mMB* complexes. Likewise, for the 4 × 10^6^ concentration under constant flow, a η_c_ of 34% was reported for the mMB-E. coli* complexes while a η_c_ of 36% was reported for the mMB* complexes. For the 2 × 10^6^ concentration under switching flow, a η_c_ of 83% was reported for the mMB-E. coli* complexes while a η_c_ of 85% was reported for the mMB* complexes. Finally, for the 4 × 10^6^ concentration under switching flow, a η_c_ of 73% was reported for the mMB-E. coli* complexes while a η_c_ of 80% was reported for the mMB* complexes. For both constant and switching cases, p > 0.05 when comparing the mMB-E. coli* results to the mMB* results. This indicates that there is no significant difference between η_c_ when working with mMB* complexes only and mMB-E. coli* complexes.

## Discussion

This study analyzes the ability of a microfluidic device to detect and quantify the concentration of mMB-E. coli* complexes in water samples. The microfluidic device was tested using mMB-E. coli* complex concentrations of 2 × 10^5^, 2 × 10^6^, 4 × 10^6^, 2 × 10^7^, and 4 × 10^7^ bacteria/mL under switching and constant flow protocols at an EOF voltage of 650 V. Fluorescent images were analyzed in order to determine a calibration curve of fluorescent intensity based on mMB-E. coli* complex concentration. Consequently, the capture efficiencies of these mMB-E. coli* complexes in the system were determined.

### Bacteria tagging

The ability to tag non-fluorescent mMBs to E. coli* is an important step for testing the operation of the device under real conditions. While the previous experiment^24^ used mMB* complexes to test the microfluidic device, this study evaluated the operation of the device under realistic scenarios by attempting to identify E. coli* in a water sample through the coupling of E. coli* with mMBs. To minimize the artifact in the device, the E. coli* must be the only fluorescent entity in the system. This study used a secondary binding antibody that would only allow E. coli* to bind to the mMBs, thus preventing the creation of fluorescent mMB (mMB*) complexes. Consequently, excess mMBs are used to ensure increased sites for coupling of mMBs with the E. coli*; thereby avoiding formation of mMB* complexes.

### Fluorescent calibration curve

Samples with mMB-E. coli* complex concentrations of 2 × 10^5^, 2 × 10^6^, 4 × 10^6^, 2 × 10^7^, and 4 × 10^7^ bacteria/mL were analyzed in the microfluidic device under an EOF voltage of 650 V using both the switching and constant flow protocols. A significant increase in fluorescent intensity is seen as the mMB-E. coli* complex concentration increases from 2 × 10^5^ to 4 × 10^7^ bacteria/mL using both the switching and constant flow protocols. More importantly, the fluorescent intensity under the switching flow protocol is significantly higher than under the constant flow protocol. This increase in fluorescence is due to the increase in residence time within the channel that experiences high magnetic field strength. The reversal of the flow in the switching protocol allows for mMBs that remained uncaptured after the first pass to return through the channel, creating the increased residence time in the channel. This allows for an increase in the number of mMB-E. coli* complexes captured and, therefore, the total fluorescence in the channel.

It is interesting to note that while the fluorescent pixel count in the calibration curve does follow a linear trend, it also exhibits a power law relationship with the exponent less than one. This levelling out of the curve could occur due to the limited confines of the channel creating overlap of fluorescent entities at higher concentrations. This would cause a dampening of the total fluorescent pixel counts at higher concentrations, creating the plateau effect seen in Fig. 6B. Similarly, the presence of additional mMBs in the system could be causing a dampening effect. This effect would become more prevalent at higher concentrations as the total area of the top channel wall (where the beads are captured) is nearing saturation. This would cause any fluorescence beneath them to be suppressed, decreasing to total fluorescent pixel count. Meanwhile, at lower concentrations, the total number of mMB-E. coli* complexes entering the system is lower, so there is a decreased likelihood that any particular fluorescent source would be blocked.

Since this curve relies on the magnetic capture of mMB-E. coli* complexes against the top wall of the channel, variations of known gas adsorption models can be used as possible fits. The Freundlich adsorption model is an empirically derived model presented in Eq. 4

4 $${q}_{e}={k}_{f}{C}_{e}^{\frac{1}{n}}$$

where q_e_ is the amount of solute adsorbed (gram adsorbate/gram adsorbent), C_e_ is the equilibrium adsorbate concentration (gram adsorbate/mL), and k_f_ and 1/n are fitting parameters. This model can be adopted to the current system by equating q_e_ to the fluorescent pixel count and C_e_ to the bacteria concentration. In this case 1/n represents the strength of the attraction to the magnet and is similar when comparing the constant and switching flow cases. Meanwhile, k_f_ represents the extent of the attraction, with the switching flow case showing a much greater value due to the returning of uncaptured beads, resulting in an increased pixel count^25–27^. The use of the Freundlich model accounts for the asymptotic trend shown in the calibration curve due to the rough surface, non-ideal interactions, and potential overlapping of mMBs and mMB-E. coli* complexes in the system. The adoption of the Freundlich model also validates the conclusions derived by the various linear trendlines shown in Fig. 6A. The trendlines show more accurate fits, as indicated by the R^2^ values closer to 1, at the lower concentration ranges compared to the higher concentration ranges. This indicates that the calibration curve is more accurate at the lower end of the concentration ranged examined while the sensitivity slightly decreases at higher concentrations.

### Capture efficiency under constant and switching flows

The η_c_ of mMB-E. coli* complexes at concentrations of 2 × 10^5^, 2 × 10^6^, 4 × 10^6^, 2 × 10^7^, and 4 × 10^7^ bacteria/mL was also determined based on fluorescent pixel analysis. The η_c_ of mMB-E. coli* complexes under switching flow were approximately 2 times higher than the capture efficiency mMB-E. coli* complexes under constant flow for all concentrations. In addition, the η_c_ for mMB* complexes only^24^ and mMB-E. coli* complexes were similar under the same flow protocols and concentrations.

### Limitations of experiment

One of the limitations of the study is that the exact concentration (or number) of mMB-E. coli* complexes captured could not be counted, instead a relative quantification of fluorescence was reported. The pixel counts based on fluorescent images of the mMB-E. coli* complexes were used to compare captured and uncaptured E. coli*, providing the η_c_ for each condition. Additionally, this study is conducted for a single voltage difference of 650 V. The η_c_ needs to be tested for different voltage differences, resulting in varied EOF values. It is likely that increasing the voltage will decrease the capture efficiency due to the increased flow rate. Also, the η_c_ needs to be optimized for different channel dimensions (length and width) and shapes. A smaller channel will likely increase capture efficiency due to a smaller maximum distance mMBs can start from the magnet. However, a smaller channel will also decrease the flow rate and number of mMB-E. coli* complexes analyzed as well as increase the potential for clogging in the channel. In addition the η_c_ was only analyzed for a stationary magnet instead of electromagnets, which are popular and may be tested for variable magnetic fields within the proposed microfluidic device. Finally, the system was only analyzed using E. coli* as the target cell of interest. It is expected that different calibration curves would be required for different bacterial entities.

### Future direction

While this study used an idealistic sample with only E. coli* for calibration purposes, the presence of other biological entities in a realistic sample needs to be further analyzed. Further optimization of the device over a wider range of bacteria concentration also needs to be addressed. The device has been shown to work well in the range considered in this study; however the plateau trend at higher concentrations (seen in Fig. 6) indicates a need to analyze higher concentrations. In addition, analyzing lower bacteria concentrations would further help calibrate the device for some specific bacteria strains such as *E. coli* O157:H7. In addition, the use of a two-step binding process within the microchannel to create a mMB-bacteria-fluorescent tag complex is necessary for the eventual application of the device. The use of two separate bacteria targeting antibodies, one for the mMB and one for the fluorescent tag, would prevent competition over binding sites on the bacteria, allowing for improved characterization and quantification of bacteria in a sample. Finally, the analysis of additional voltages between 100–900 V, where Joule heating is at a minimum, needs to be analyzed in order to optimize capture efficiency and accuracy of the device.

## Data Availability

The datasets generated during and/or analyzed during the current study are available from the corresponding author on reasonable request.
